# The Treatment of Harelip and Cleft Palate

**Published:** 1899-04

**Authors:** 


### The Treatment of Harelip and Cleft Palate.

This much discussed topic continues to be the subject of a good deal of doubt in many minds as to when and how to operate for the various conditions that present themselves. Many of the procedures necessary are entirely within the range of the general practitioner, but there always remains a feeling of hesitation as to the methods most advisable to employ, and the most suitable time for operation. Towards solving such doubts an authoritative review of the recent literature of the subject, and conclusive statements as to what seems best in the therapeutic suggestions that have been recently offered by various writers will be of the greatest value to the busy practitioner.

Such a review of the treatment of Harelip and Cleft Palate is given by Dr. J. Chalmers DaCosta, in "*Progressive Medicine,*"* the new quarterly review of advances in medicine, of which Professor Hare is the editor. From it we gather that the tendency is more and more towards early operation. The third or fourth month used to be considered the earliest suitable time to operate. Murray now counsels operation in the fourth week; Mumford and Heath think it should be undertaken not later than from the sixth to the eighth week. Where cleft palate exists it is not operated upon so early. The harelip is operated upon alone, and the persistent pressure made by the closed lips helps to lessen the gap in the growing bone. The operation on the cleft palate is put off for a while, but this, too, not nearly so long as it used to be. If the closure of the defect is

*"Progressive Medicine". a Quarterly Digest of New Methods, Discoveries, and Improvements in the Medical and Surgical Sciences. Volume I, No. 1, March, 1899. Edited by Hobart A. Hare, M. D. Lea Brothers & Co. New York and Philadelphia.

delayed until the child has learned to talk, the peculiarities of speech, especially its offensive nasal character, will never be corrected. The authorities are agreed, then, that a cleft in the soft palate should be closed about the sixth month, and in the hard palate during the second year.

The practical suggestions collected from the recent literature of the subject by Dr. DaCosta are very valuable to the ordinary practitioner. Space will permit us to give but a few of them. The use of the knife in operation rather than the scissors, because the latter crushes tissues more, leaving its vitality impaired, especially at the edges where this is so important for subsequent union; the avoidance of pins or heavy sutures in securing proper apposition after the operation is advised, though these are faults of technique in this matter that we fear have been so ground into the present generation by text-book and teacher that failures of union due to these crude early methods will still continue to be frequent. The suggestion by Mumford as to anchoring the nares with shotted wire will remove a very common cause of failure due to the child's inevitable tendency to "turn up its nose" at and after the proceedings.

In double harelip it is advised to remove the intermaxillary bone by sub-periosteal operation a week before the operation on the lip. If left it is liable to undergo necrosis. Its removal leads to some flattening, but this will not be great if the bone be removed by subperiosteal operation, and if but one side of the harelip be operated upon at a time. Among the directions for the operation for cleft of the hard palate, we note these pre-operative measures of precaution from Owen, which are sometimes forgotten, but of which the practical value it is easy to see: never operate unless the child is in the best possible health; remove carious teeth, adenoids and enlarged tonsils before operating, and operate whenever possible in fine weather, so that the patient can get out of doors soon afterwards. The neglect to remove such ready sources of infection as carious teeth and those harborers of microbes, the irresistive tissues of adenoids and enlarged tonsils, is very probably the source of a good many of the failures in uranoplastic osteo resection.

